# High-Fat Diet Induces Dysbiosis of Gastric Microbiota Prior to Gut Microbiota in Association With Metabolic Disorders in Mice

**DOI:** 10.3389/fmicb.2018.00639

**Published:** 2018-04-09

**Authors:** Cong He, Dandan Cheng, Chao Peng, Yanshu Li, Yin Zhu, Nonghua Lu

**Affiliations:** ^1^Department of Gastroenterology, The First Affiliated Hospital of Nanchang University, Nanchang, China; ^2^Jiangxi Supervision and Inspection Center for Medical Devices, Nanchang, China

**Keywords:** high-fat diet, gastric microbiota, gut microbiota, metabolic disorders, 16S rRNA gene sequencing

## Abstract

Accumulating evidence suggests that high-fat diet (HFD) induced metabolic disorders are associated with dysbiosis of gut microbiota. However, no study has explored the effect of HFD on the gastric microbiota. This study established the HFD animal model to determine the impact of HFD on the gastric microbiota and its relationship with the alterations of gut microbiota. A total of 40 male C57BL/6 mice were randomly allocated to receive a standard chow diet (CD) or HFD for 12 weeks (12CD group and 12HFD group) and 24 weeks (24CD group and 24HFD group) (*n* = 10 mice per group). Body weight and length were measured and Lee’s index was calculated at different time points. The insulin sensitivity and serum levels of metabolic parameters including blood glucose, insulin and lipid were also evaluated. The gastric mucosa and fecal microbiota of mice were characterized by 16S rRNA gene sequencing. The body weight was much heavier and the Lee’s index was higher in 24HFD group than 12HFD. The insulin resistance and serum level of lipid were increased in 24HFD group compared to 12HFD, indicating the aggravation of metabolic disorders as HFD went on. 16S rRNA gene sequencing showed dysbiosis of gastric microbiota with decreased community diversity while no significant alteration in gut microbiota after 12 weeks of HFD. The phyla Firmicutes and Proteobacteria tended to increase whereas Bacteroidetes and Verrucomicrobia decrease in the gastric microbiota of 12HFD mice compared to 12CD. Moreover, a remarkable reduction of bacteria especially *Akkermansia muciniphila*, which has beneficial effects on host metabolism, was observed firstly in the stomach of 12HFD group and then in the gut of 24HFD group, indicating the earlier alterations of microbiota in stomach than gut after HFD. We also found structural segregation of microbiota in the stomach as well as gut between 12HFD and 24HFD group, which is accompanied by the aggregation of metabolic disorders. These data suggest that HFD affects not only gut microbiota but also gastric microbiota and the disruption of microbial ecosystem in the digestive tract may play a part in the development and progression of metabolic diseases although molecular mechanism requires further investigation.

## Introduction

With the rapid development of the economy, secondary lifestyles and the intake of a high-fat diet have increased dramatically, leading to the prevalence of overweight and obesity worldwide. Obesity is the excess or abnormal accumulation of fat or adipose tissue in the body that may impair health. It is the most common risk factor for metabolic disorders such as type 2 diabetes, cardiovascular disease, fatty liver and even cancer. Obesity has become an epidemic that has worsened over the last 50 years and is the second most common cause of preventable death after smoking (Panuganti and Lenehan, 2017). Consequently, preventive and therapeutic strategies to reduce the morbidity and mortality caused by obesity are important.

The gut harbors the greatest density of microbes in the human body (more than 100 trillion, with a total weight of up to 1.5 kg); these microbes play an important role in energy regulation, vitamin production and nutrient harvest (Sender et al., 2016; Maruvada et al., 2017). Recent mounting evidence suggests that the gut microbiota is involved in the development of obesity in animals and humans (Zhao, 2013). For example, obese humans and rodents have an altered gut microbiota composition with less phylogenic diversity than that of lean controls, and transplantation of the gut microbiota from obese subjects to germ-free mice can transfer the obese phenotype (Turnbaugh et al., 2006; Ridaura et al., 2013). Furthermore, germ-free mice are leaner than conventionally raised mice and are protected against diet-induced obesity, which further verifies the causal relationship between gut microbiota and obesity (Backhed et al., 2007).

The normal human stomach was long thought to be sterile, due largely to the gastric acid barrier until the discovery of *Helicobacter pylori*. The gastric microbiota has traditionally been identified at relatively low abundance by cultivation of gastric juice or mucosa biopsies. To date, with the development of new nucleotide sequencing techniques and bioinformatics tools the diversity and complexity of the microbiota in the stomach have been recognized (He et al., 2016). The gastric bacterial rDNA data-set significantly differed from sequence collections of the human mouth and esophagus, indicating that the human stomach may be home to a distinct microbial ecosystem. Recent accumulating evidence suggests that microbial dysbiosis in the stomach may play a role in the development of gastric cancer (Ferreira et al., 2017; Shah, 2017). However, the distribution of the gastric microbiome in obesity and metabolic diseases remains largely unknown. Therefore, we explored the effects of high-fat diet on both gastric and gut microbiota in C57BL/6 mice by high throughput sequencing and determined the relationship between changes in bacteria at different anatomic locations and the development of metabolic disorders.

## Materials and Methods

### Animals and Diets

Seven- to eight-week-old male C57BL/6 mice (Hunan Slac Jingda Laboratory Animal Company, Ltd., Changsha, China) with similar initial body weight were housed in a controlled environment (12 h per day/night cycle and lights off at 19:00) with free access to food and water. All animal protocols were approved by the Institute of Animal Care and Use Committee (Approval Nos. SCXK 2013-0004 and 43004700006655). The animal experiments were performed in accordance with the Guidelines for the Care and Use of Laboratory Animals at The First Affiliated Hospital of Nanchang University. As it has been reported that metabolic disorders could be successfully induced after 12 weeks of high fat diet in C57BL/6 mice, we chose 12 and 24 weeks for study observation (Xu et al., 2016). After 1 week of acclimation on a normal chow diet, we randomly assigned the animals to four groups (10 mice per group) with either a standard chow diet (CD) (12.04% of energy from fat, Beijing KeAo XieLi Company, Ltd., Beijing, China) or a high-fat diet (HFD) (45.37% of energy from fat, Beijing KeAo XieLi Company, Ltd., Beijing, China) for 12 and 24 weeks. Animal weights and lengths (nasal-anal lengths) were monitored using an electronic precision balance and a ruler at different time points. To determine the body mass index of mice, Lee’s index was used and calculated by the formula [body weight (g) × 1000/body length (cm)]^1/3^.

### Glucose Homeostasis

After 12 and 24 weeks on different diets, mice were fasted overnight, and the blood glucose levels in the tail vein were measured using a handheld glucometer (OneTouch Ultra Easy, LifeScan). For the intraperitoneal glucose tolerance test (IPGTT), glucose concentrations were measured at 15, 30, 60, and 120 min after intraperitoneal injection of a glucose load (2 g/kg). For the intraperitoneal insulin tolerance test (IPITT), mice were fasted for 6 h, after which 0.75 U/kg body weight of insulin (Novolin R, Novo Nordisk, Copenhagen, Denmark) was injected intraperitoneally, and blood glucose levels were determined as above at 0, 15, 30, 60, and 120 min.

### Blood Serum Analysis

At weeks 12 and 24, mice were fasted overnight and sacrificed for subsequent analysis. Blood was collected in microfuge tubes and allowed to clot for 30 min. Then, samples were centrifuged at 3000 rpm for 20 min, and serum was collected and stored at -80°C until analysis. Serum insulin (Crystal Chem Inc.) was quantified by ELISA. The homeostasis model assessment of insulin resistance (HOMA-IR) index was calculated using the formula [fasting insulin (μUI/ml) × fasting glucose (mM)/22.5]. Serum triglycerides (TG), total cholesterol (TC), high-density lipoprotein (HDL-C) and low-density lipoprotein (LDL-C) of the mice were detected by an automatic biochemical analyzer (OLYMPUS AU5421).

### 16S rRNA Gene Sequencing

Fresh fecal and gastric mucosa samples were randomly collected from half of the animals in each group (*n* = 5 per group). The stomach of each mice was aseptically removed and incised along the greater curvature. Samples subjected to microbiota analysis included three sections from each stomach (fundus, antrum, and corpus; these steps were under sterile conditions) and snap-frozen. DNA extraction was performed as previously described (Bik et al., 2006). Total genomic DNA was extracted using the QIAamp DNA isolation kit (Qiagen) following the modified protocol for Gram-positive bacteria combined with a bead-beating step (Lofgren et al., 2011). The concentration and integrity of bacterial DNA were assessed using a Nanodrop (Thermo Scientific) and agarose gel electrophoresis, respectively. The 16S rRNA gene amplicon sequencing was performed on the Illumina MiSeq platform using universal primers 515f, 5′-GTGCCAGCMGCCGCGGTAA-3′; and 806r, 5′-GGACTACHVGGGTWTCTAAT-3′ targeting the V4 hypervariable regions.

### Microbial Bioinformatic Analysis

The raw data were filtered to obtain clean reads by eliminating the adapter pollution and low-quality sequences (Trimmomatic, version 0.30^1^), and the reads were truncated at any site receiving an average quality score < 20 over a 50 bp sliding window. High- quality paired-end reads were combined with tags with an average read length of 252 bp using FLASH (Fast Length Adjustment of Short reads, v 1.2.11). The reads were then clustered as OTUs (Operational Taxonomic Units) by scripts of USEARCH (version 7.1) software with a 97% similarity threshold. Chimeric sequences were identified and deleted. The representative OTU sequences were taxonomically classified using Ribosomal Database Project (RDP) Classifier (version 2.2) against the Silva (SSU123) 16S rRNA database. Finally, an OTU table and a phylogenetic tree were generated for diversity analysis. To estimate the diversity of the microbial community of the sample, we calculated the within-sample (α) diversity based on the genera profiles. β diversity was estimated by computing Bray–Curtis distance and was visualized with principal coordinate analysis (PCoA). PLS-DA (Partial Least Squares-Discriminant Analysis) was performed using the R package to cluster the sample plots across groups. Differential abundance of phyla, genera and KEGG (Kyoto Encyclopedia of Genes and Genomes) pathways were tested by the Wilcoxon rank sum test. Linear discrimination analysis coupled with effect size (LEfSe) was performed to identify the bacterial taxa differentially represented between groups at the genus or higher taxonomy levels (Segata et al., 2011).

### Statistical Analysis

For analyzing the effect of HFD on the gastric (G) and gut (F) microbiota after 12 and 24 weeks, some groups were compared: 12CD_G/12HFD_G, 12CD_F/12HFD_F, 24CD_G/24HFD_G, and 24CD_F/24HFD_F. Differences in bacterial communities of mice fed a HFD for 12 weeks vs. 24 weeks were identified by comparing 12HFD_G/24HFD_G and 12HFD_F/24HFD_F. Data are expressed as the mean ± standard error of mean (SEM). The differences between groups with normal distribution were evaluated by Student’s *t*-test. Statistical analysis was performed using the SPSS 13.0 software and was considered to be statistically significant if *p* < 0.05.

### Accession Number

The sequences have been deposited in the NCBI Sequence Read Archive Database under accession number SRP132758.

## Results

### Long-Term High-Fat Diet Induces More Severe Metabolic Disorders Than Does Short-Term High-Fat Diet in Mice

First, we compared the effects of HFD on body parameters at different time points in mice. As shown in **Table 1**, the body weight was significantly increased after 12 weeks of feeding a HFD and continued to rise at 24 weeks. Moreover, the HFD-fed mice at 24 weeks were much heavier than those at 12 weeks (*p* < 0.05). The difference in Lee’s index, which reveals the obesity of mice, between the CD and HFD groups was more remarkable at 24 weeks than at 12 weeks. The Lee’s index of HFD-fed mice at 24 weeks tended to be higher than that at 12 weeks (*p* = 0.06).

**Table 1:** The metabolic parameters of mice given chow diet (CD) or high fat diet (HFD) after 12 and 24 weeks, respectively.

	12 weeks			24 weeks		
	CD	HFD	*p*-value	CD	HFD	*p*-value
Body weight	27.6 ± 1.9	33.3 ± 2.6	<0.001	28.9 ± 1.6	42.1 ± 5.3	<0.001
Lee’s index	329.8 ± 6.0	339.2 ± 9.4	0.016	317.4 ± 9.9	350.9 ± 15.9	<0.001
FBG	5.8 ± 0.9	8.3 ± 1.4	<0.001	6.0 ± 1.5	8.4 ± 1.6	0.002
Serum insulin	0.4 ± 0.2	0.5 ± 0.3	0.218	0.6 ± 0.2	1.0 ± 0.4	0.005
HOMA-IR	2.6 ± 1.5	4.7 ± 2.4	0.028	4.6 ± 1.7	9.7 ± 2.8	<0.001
TG	0.5 ± 0.2	0.6 ± 0.2	0.416	0.5 ± 0.4	0.4 ± 0.2	0.402
TC	3.0 ± 0.5	4.6 ± 0.4	<0.001	2.8 ± 0.4	4.9 ± 0.6	<0.001
LDL-C	0.27 ± 0.07	0.42 ± 0.08	0.001	0.22 ± 0.05	0.65 ± 0.17	<0.001
HDL-C	2.48 ± 0.41	3.76 ± 0.28	<0.001	2.24 ± 0.39	3.44 ± 0.34	<0.001
LDL/HDL	0.1 ± 0.03	0.1 ± 0.02	0.947	0.1 ± 0.02	0.2 ± 0.04	<0.001
ITT AUC	486.3 ± 89.1	547.9 ± 98.8	0.143	527.2 ± 110.3	870.9 ± 266.0	<0.001
GTT AUC	1631.0 ± 256.1	2412.1 ± 434.3	<0.001	1690.3 ± 233.6	2479.5 ± 309.3	<0.001

*n = 10 mice/group. Data are expressed as the mean ± SEM. FBG, fasting blood glucose; HOMA-IR, homeostasis model assessment of insulin resistance index; TG, triglycerides; TC, total cholesterol; HDL-C, high-density lipoprotein; LDL-C, low-density lipoprotein; ITT, insulin tolerance test; GTT, glucose tolerance test; AUC, area under curve.*

We next examined the impact of HFD duration on glucose homeostasis and insulin sensitivity in mice. We found that the HFD group at 24 weeks displayed more severe insulin resistance than at 12 weeks as revealed by IPITT (*p* < 0.05). The fasting blood glucose (FBG) was already markedly elevated in HFD-fed mice compared with CD-fed mice after 12 weeks, while the serum insulin level was statistically increased after 24 weeks of feeding a HFD (**Table 1**). Although the glucose intolerance was not different in the HFD groups between 12 and 24 weeks, the higher fasting hyperinsulinemia and HOMA-IR index values further suggested that the disruption of glucose metabolism was more severe after long-term HFD (*p* < 0.05).

To evaluate whole-body lipid homeostasis, we measured the serum levels of biochemical parameters in mice at different groups. HFD significantly increased TC, including LDL-C and HDL-C, without affecting the TG level (**Table 1**). Consistently, the long-term HFD was likely to aggravate dyslipidemia compared to short-term HFD, as revealed by a higher level of LDL-C and LDL-C/HDL-C as well as a lower level of HDL-C (*p* < 0.05).

### The Alteration of Gastrointestinal Microbiota After Short-Term HFD

**FIGURE 1:**
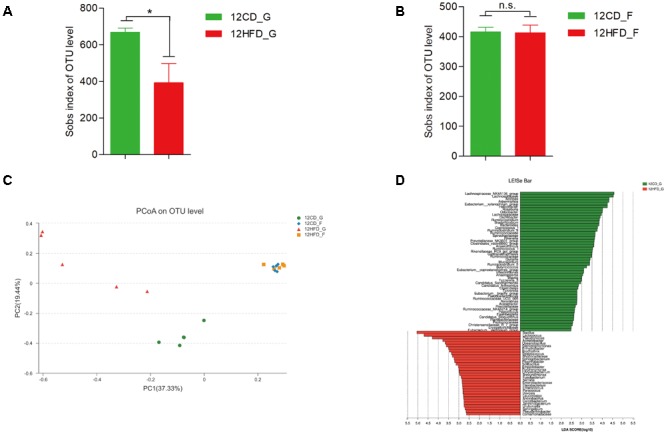
The impact of HFD on the composition of gastric (G) and gut (F) microbiota in mice after 12 weeks (*n* = 5 for each group). Decreased alpha diversity in 12HFD_G compared with 12CD_G **(A)** while no significant difference between 12HFD_F and 12CD_F **(B)** (^∗^*p* < 0.05). Principal coordinate analysis (PCoA) of bacterial beta diversity based on the Bray-Curtis dissimilarity **(C)**. **(D)** Linear discriminant analysis (LDA) coupled with effect size measurements identified the most differentially abundant taxa between 12HFD_G and 12CD_G. 12HFD_G enriched taxa were indicated in red while taxa enriched in 12CD_G were represented in green. Only taxa meeting an LDA significant threshold of >2 were shown.

The 16S rRNA gene sequencing of gastric mucosa samples revealed that the observed species and the two indexes reflecting species richness and diversity (Chao index and Shannon index, respectively) were reduced in 12HFD_G compared with 12CD_G (**Figure 1A** and **Supplementary Figures S1A,B**), while there was no significant difference in alpha diversity between 12CD_F and 12HFD_F (**Figure 1B** and **Supplementary Figures S1C,D**). The Bray–Curtis based principal coordinates analysis (PCoA) at the OTU level showed that the gastric microbiota was separated clearly from that in the gut (**Figure 1C**, Adonis analysis, *p* = 0.006). Moreover, the 12HFD_G group displayed a distinct microbiota community that clustered separately from the 12CD_G, while the 12CD_F and 12HFD_F groups were not separated into different clusters (**Figure 1C**, Adonis analysis, *p* = 0.003 for gastric samples and *p* = 0.327 for fecal samples). Compared to the 12CD_G group at the phylum level, the 12HFD_G tended to have a higher proportion of sequences assigned to Firmicutes and Proteobacteria, whereas reads assigned to Bacteroidetes and Verrucomicrobia were relatively less frequent (**Supplementary Figure S2**). To identify bacterial taxa that significantly differentiated between 12CD_G and 12HFD_G, a metagenomic biomarker discovery approach (LEfSe) was used. We found that *Streptococcus, Enterococcus*, and an unclassified member of *Enterobacteriaceae* were significantly enriched in the 12HFD_G group, while sequences from *Akkermansia* spp. were more abundant in 12CD_G (**Figure 1D**).

### The Alteration of Gastrointestinal Microbiota After Long-Term HFD

**FIGURE 2:**
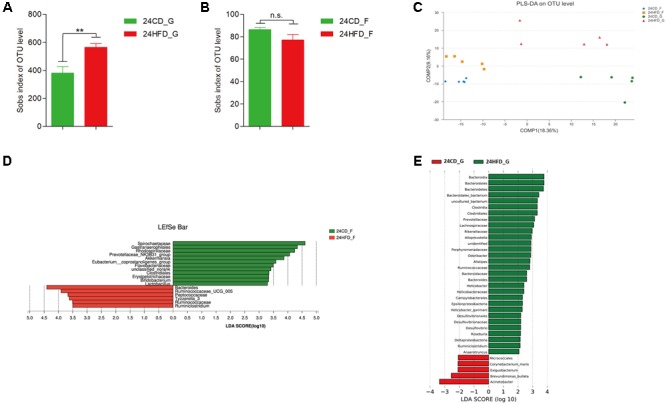
The impact of HFD on the composition of gastric (G) and gut (F) microbiota in mice after 24 weeks (*n* = 5 for each group). Alpha diversity was increased in 24HFD_G compared with 24CD_G **(A)**, while no significant difference was observed between 24HFD_F and 24CD_F **(B)** (^∗∗^*p* < 0.01). **(C)** Partial least-squares discriminant analysis (PLS-DA) scores plot showed apparent distinctions between 24HFD_G (red) and 24CD_G (green) as well as 24HFD_F (yellow) and 24CD_F (blue). LEfSe analysis identified the most differentially abundant taxa between 24CD_F and 24HFD_F **(D)** as well as 24CD_G and 24HFD_G **(E)**. Only taxa meeting an LDA significant threshold of >2 were shown.

The number of observed species reflecting alpha diversity increased in 24HFD_G compared to that in 24CD_G, while there was no significant difference between 24CD_F and 24HFD_F (**Figures 2A,B**). The PLS-DA showed a significant separation between 24HFD_F and 24CD_F groups as well as 24HFD_G and 24CD_G groups (**Figure 2C**). In addition, the microbiota composition from gastric mucosa and fecal samples was separated indicating there may be a distinct bacterial community at different sites of the body (Adonis analysis, *p* = 0.007). At the genus level, the 24HFD_F had lower abundance of potential beneficial bacteria such as *Akkermansia, Bifidobacterium*, and *Lactobacillus* than did 24CD_F, while the 24HFD_G had higher abundance of *Lachnospiraceae, Rikenellaceae*, and *Desulfovibrio*, which have been reported to be positively correlated with obesity, than did 24CD_G (Zhang et al., 2010) (**Figures 2D,E**).

### The Long-Term HFD Promoted the Dysbiosis of Gastrointestinal Microbiota Induced by Short-Term HFD

**FIGURE 3:**
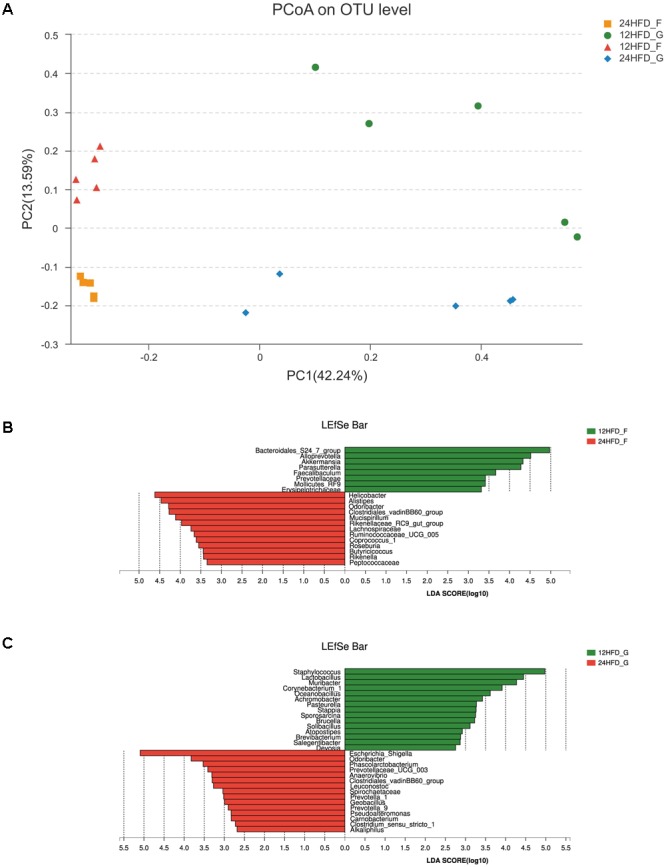
The alteration of gastric (G) and gut (F) microbiota in mice fed HFD after different periods. **(A)** Beta diversity as revealed by PCoA analysis based on the Bray–Curtis distance showed that the samples of 12 weeks (12HFD_G and 12HFD_F) clustered separately from those of 24 weeks (24HFD_G and 24HFD_F). LEfSe analysis was used to identify the most differentially abundant taxa between 12HFD_F and 24HFD_F **(B)** as well as 12HFD_G and 24HFD_G **(C)**. The enriched taxa in mice given HFD for 24 weeks were indicated in red while taxa enriched in those given HFD for 12 weeks were represented in green. Only taxa meeting an LDA significant threshold of >2 were shown.

As shown above, metabolic syndrome (MS) including obesity, insulin resistance and hyperlipemia was more severe in mice after 24 weeks HFD than that at 12 weeks; thus, we wonder whether the aggravation of MS was associated with changes in gastrointestinal microbiota. The Bray–Curtis based PCoA revealed that the gastric mucosa samples (12HFD_G, 24HFD_G) clustered separately from the fecal samples (12HFD_F, 24HFD_F), and the samples from 24 weeks (24HFD_G, 24HFD_F) also showed a clear separation from those from 12 weeks (12HFD_G, 12HFD_F) (**Figure 3A**). There was no significant alteration of alpha diversity as reflected by observed species between samples from different time points in both the stomach and gut (**Supplementary Figure S3**). Using the LEfSe method, several microbial signatures in the gastric and gut microbiota were different between 12 and 24 weeks. At the genus level, the relative abundance of *Bacteroidales_S24-7_group* and *Akkermansia*, which were reported to have an anti-obesity effect, was markedly decreased in the 24HFD_F compared to that in 12HFD_F (**Figure 3B**). Additionally, the relative abundance of *Lactobacillus* was enriched in 12HFD_G, while the potential pathogenic bacterium *Escherichia_Shigella* was prevalent in 24HFD_G (**Figure 3C**).

## Discussion

The intestinal microbiota has been extensively studied at both the phylogenetic and metagenomic levels in the context of metabolic disorders (Kim et al., 2012; Moreira et al., 2012; Carmody et al., 2015). The novelty of the present work lies in the comprehensive characterization of gastric microbial communities in the mice after different periods of feeding a HFD and their correlation with alterations of gut microbiota in the development and progression of metabolic syndrome.

The gastric environment had long been considered bacteria-free until the discovery of *H. pylori* in 1983 (Marshall and Warren, 1984). Using culture-independent methods of analysis, evolving data show that *H. pylori* is not the only inhabitant of the gastric mucosa. Since the first internal biogeographic map of the human stomach was completed by Bik et al. (2006), the disruption of human gastric microbiota has been identified as a trigger of gastric diseases, especially gastric cancer (Petra et al., 2017). However, the changes in gastric microbiota in metabolic disorders remain largely unknown. Herein, we conducted 16S rRNA gene sequencing to characterize the bacterial profile in the stomach of HFD-fed mice. After feeding a HFD for 12 weeks, the metabolic parameters such as body weight, Lee’s index, fasting blood glucose etc. were significantly higher than those in the controls, indicating the successful establishment of the animal model with metabolic diseases. Both the alpha and beta diversity analyses showed that the composition of gastric microbiota in the HFD group at 12 weeks was distinctly different from that of the CD group without changes in gut microbiota, which suggests that the gastric microbiota may be more sensitive to a HFD than the gut microbiota. In addition to the gut, the stomach plays an important role in food digestion, and its direct contact with dietary ingredients could predispose the impairment of gastric microbiota (Bornhorst et al., 2016). Besides, the resistance of gut microbiota to the effect of short-term HFD is probably due to its larger bacterial community and stronger self-regulating ability, while the gastric microbiota structure is relatively simple and thus more susceptible (Tap et al., 2015).

At the phylum level, we observed that the ratio of Firmicutes/Bacteroidetes tended to increase in the stomach of HFD-fed mice compared to the controls at 12 weeks, and this compositional reshape in the microbial community has been thought to be a typical characteristic of obesity-driven dysbiosis in both humans and animals (Mozes et al., 2008; Cotillard et al., 2013). Moreover, mice fed a HFD for 12 weeks had a decreased percentage of *Akkermansia* spp., which was mostly attributed to a significant reduction of *Akkermansia muciniphila* (*A. muciniphila*) in the stomach. Recently, *A. muciniphila* has been considered as a novel functional microbe with probiotic properties in host metabolism (Derrien et al., 2017). In clinical studies, the abundance of *Akkermansia* generally decreased in individuals with metabolic impairments, such as obese children and adults (Karlsson et al., 2012; Teixeira et al., 2013). Others showed negative correlations between *Akkermansia* spp. and markers of metabolic disorder (Escobar et al., 2014; Remely et al., 2015). While these studies were indicative of an association with *Akkermansia*, some interventional studies have provided direct support for the role of *A. muciniphila* in preventing metabolic disorders. It was found that the daily administration of *A. muciniphila* could counteract the deleterious metabolic features induced by a HFD in mice (Everard et al., 2013). Taken together, these studies noted the beneficial role of this bacteria in the gut, and our findings further suggest that its abundance in the stomach is also negatively associated with metabolic features.

In contrast to the reduction of *Akkermansia*, several bacteria such as *Streptococcus, Enterococcus* and an unclassified member of *Enterobacteriaceae*, which are normally prevalent in the gut, were enriched in HFD-fed mice at 12 weeks. Consistent with previous studies, these bacteria have been reported as positively correlated with the development of metabolic diseases and their enrichment in the stomach may be harmful (Karlsson et al., 2012; Jie et al., 2017; Korpela et al., 2017). The overgrowth of *Enterobacteriaceae* induced by a HFD increased the production of endotoxin, which could further trigger chronic inflammation and thereby accelerate obesity (Kim et al., 2012). In our opinion, the alterations of gastric microbiota may due to the abnormal secretion of gastric acid, which is probably the most important factor for bacteria colonization in the stomach. Indeed, gastric acidity (pH < 4) is generally considered to be an effective barrier to microbial overgrowth, and the acid secretion by the gastric parietal cell is regulated by paracrine, endocrine and neural pathways (Wu et al., 2014). Recently, a study by Chen et al. (2016) revealed a dominant connection between the stomach and obesity in two obese mouse models, namely, ob/ob and HFD fed mice. They found the differential expression of genes that had known associations with obesity, diabetes and insulin resistance in the stomach of obese mice. Furthermore, the gene expression profiles were strongly associated with increased gastric acid secretion probably through activation of the gastrin pathway. Taken together, we speculate that a HFD could promote the production of gastrin and then stimulate the secretion of gastric acid, which in turn could induce the dysbiosis of gastric microbiota, including reduction of beneficial taxa and enrichment of potential pathogenic bacteria after 12 weeks.

With the extension of the HFD, the gut microbiota community of the HFD group at 24 weeks clustered separately from that of the CD group as revealed by PLS-DA analysis, although no significant difference between these two groups was observed at 12 weeks. Consistently, the effect of a HFD on the gut microbiota has been studied extensively in animals and a HFD caused shifts in the diversity and taxa distribution of gut bacterial communities (Daniel et al., 2014). The LEfSe analysis, which identified the characteristic taxa present in each group, showed that the beneficial bacteria such as *Akkermansia, Bifidobacterium*, and *Lactobacillus* were remarkably reduced in the gut of HFD-fed mice after 24 weeks. Interestingly, the probiotic bacterium *Akkermansia*, as mentioned above, decreased in the gut of HFD-fed mice, which was subsequent to the reduction in the stomach, indicating that dysbiosis of gastric microbiota occurred earlier than in the gut after HFD stimulation. Additionally, the low abundance of *Bifidobacterium* has been reported to be associated with diet induced obesity, and supplementing with this bacterium in obese mice resulted in a significant reduction of body and organ weights, improved serum of levels of glucose, triglyceride, and cholesterol (Cani et al., 2007; Ray et al., 2018). Besides, *Lactobacillus* has also been reported to exert an anti-obese effect in diet-induced obesity murine models, and the possible mechanisms may involve the maintenance of the intestinal barrier and protection from chronic inflammation (Naito et al., 2011). Similar to that observed at 12 weeks, the composition of gastric microbiota in the HFD group at 24 weeks remained distinctly different from that in the CD group, which suggests the permanent effect of HFD on gastric microbiota. Consistent with previous studies, we found an increase in the abundance of gastric microbiota such as *Rikenellaceae* and *Lachnospiraceae*, which have been reported to be positively correlated with epididymal adipose tissue weight (Higashimura et al., 2017). The enrichment of *Desulfovibrio*, which belongs to the family *Desulfovibrionaceae*, was also observed in the stomach of HFD-fed mice. A study by Xiao et al. (2014) demonstrated that *Desulfovibrionaceae* was potentially an important group of endotoxin producers that could produce lipopolysaccharides (LPS) and induce chronic inflammation and metabolic endotoxemia (Xiao et al., 2014).

As expected, the metabolic parameters including body weight, insulin resistance and serum lipid were higher in mice fed a HFD for 24 weeks than those fed a HFD for 12 weeks, which indicated that the longer the HFD duration was, the more severe the metabolic disorders. Concurrent with the disturbance of metabolic phenotype, the composition of gastric as well as gut microbiota was dramatically different between the 12- and 24-week HFD groups. Intriguingly, the bacterium *Akkermansia* was once again identified as one of the taxa differentially represented between fecal samples from the 12- and 24-week HFD groups. According to the probiotic properties of *Akkermansia* in the host metabolism, its reduction in the gut in the 24-week HFD group compared to that in the 12-week HFD group may be associated with the aggravation of metabolic disorders. In addition to *Akkermansia*, the relative abundance of *Bacteroidales_S24_7_group*, which was reported to have an effect of relieving HFD-induced obesity, was also reduced. A study by Zhao et al. (2017) reported that a combination of quercetin and resveratrol restored the gut microbiota dysbiosis induced by a HFD with markedly increased *Bacteroidales_S24_7_group* and *Akkermansia*, and ameliorated HFD-induced obesity (Zhao et al., 2017). Besides, LEfSe analysis of gastric microbiota showed the reduction of beneficial *Lactobacillus* and enrichment of opportunistic pathogen *Escherichia_Shigella* in the HFD-fed mice at 24 weeks compared to 12 weeks. In total, our findings suggest that not only the gut microbiota but also the gastric microbiota may play a role in the aggravation of metabolic disturbance, although the molecular mechanism requires further investigation.

## Conclusion

We investigated the impact of a HFD on the gastric and gut microbiota after different periods of time. The results showed that the composition of the gastric microbiota changed earlier than did that of the gut microbiota, which suggests that the former is more sensitive to a HFD. Furthermore, we found that the microbiota structure in both the stomach and gut was distinctly different between long- term HFD and short-term HFD which was accompanied by the deterioration of metabolic disorders. The representative bacterium altered throughout this process was probiotic *A. muciniphila*, which was reduced in the stomach prior to the gut after short-term HFD and decreased further after long-term HFD. Thus, our study indicated that a HFD affects not only gut microbiota but also gastric microbiota, and the alterations of gastrointestinal microbiota are associated with both the development and the progression of metabolic diseases.

## Author Contributions

NL gave the direction of the paper’s conception. DC, CP, and YL performed the experiments. CH analyzed the sequence data and wrote the paper. YZ provided the intellectual support and modified language. All authors read and approved the final manuscript for publication.

## Conflict of Interest Statement

The authors declare that the research was conducted in the absence of any commercial or financial relationships that could be construed as a potential conflict of interest.

## Footnotes

1 http://www.usadellab.org/cms/?page=trimmomatic

## Supplementary Material

FIGURE S1 Alpha diversity as revealed by Chao and Shannon index were reduced in 12HFD_G compared with 12CD_G **(A,B)** (^∗^*p* < 0.05, ^∗∗^*p* < 0.01), while no significant difference was observed between 12HFD_F and 12CD_F **(C,D)**.

FIGURE S2 Bacterial taxonomic profiling at the phylum level of gastrointestinal bacteria from different groups after 12 weeks.

FIGURE S3 Alpha diversity as revealed by numbers of observed operational taxonomic units (OTUs) was not significant between 12HFD_G and 24HFD_G **(A)** as well as 12HFD_F and 24HFD_F **(B)**.
