# Supplementary figures and images for: High-Fat Diet Induces Dysbiosis of Gastric Microbiota Prior to Gut Microbiota in Association With Metabolic Disorders in Mice
Source: Front Microbiol. 2018 Apr 9;9:639. doi: 10.3389/fmicb.2018.00639 (PMC5900050; doi:10.3389/fmicb.2018.00639)

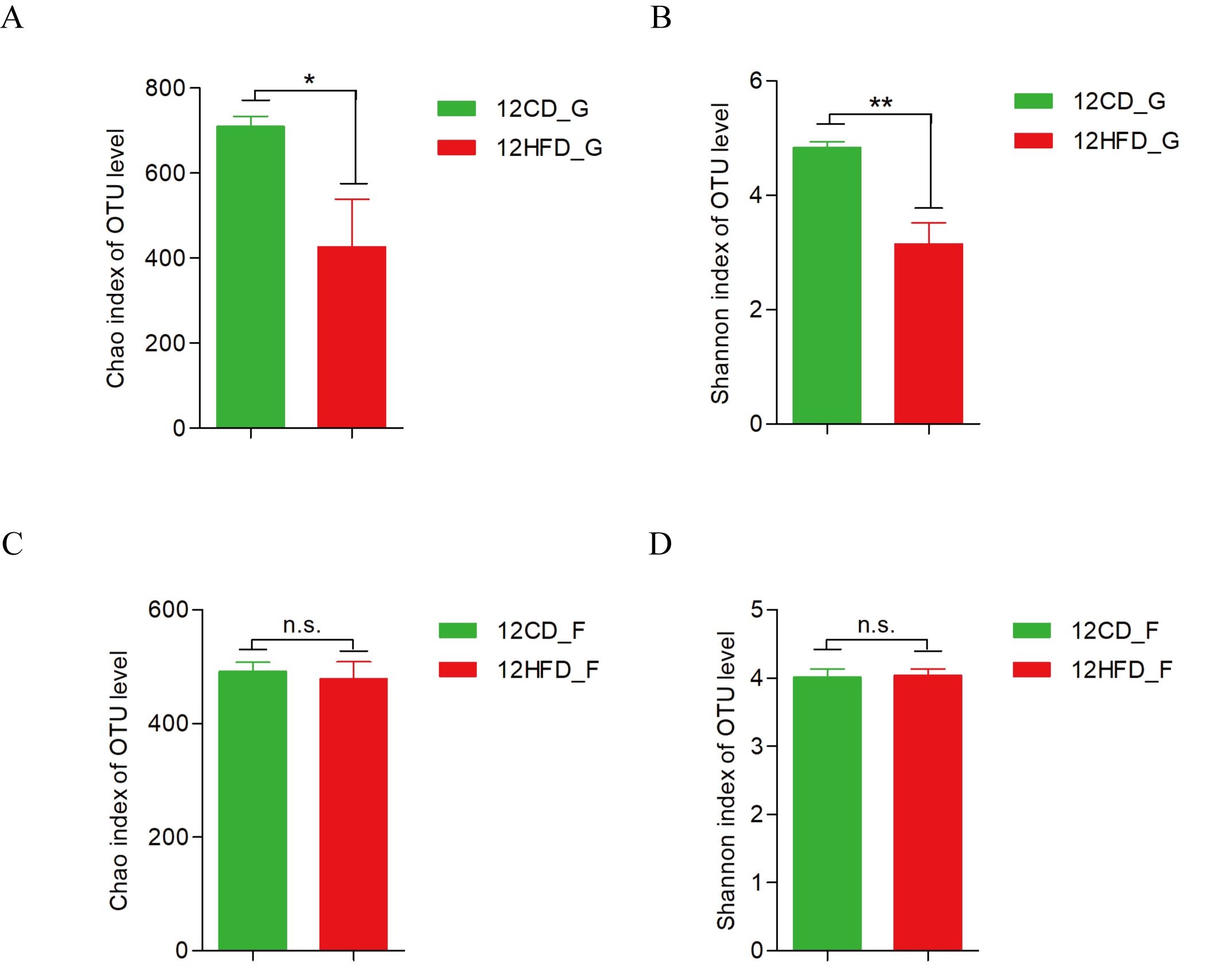

Supplement: FIGURE S1 — Alpha diversity as revealed by Chao and Shannon index were reduced in 12HFD_G compared with 12CD_G (A,B) (∗p < 0.05, ∗∗p < 0.01), while no significant difference was observed between 12HFD_F and 12CD_F (C,D). [file Image_1.TIF]

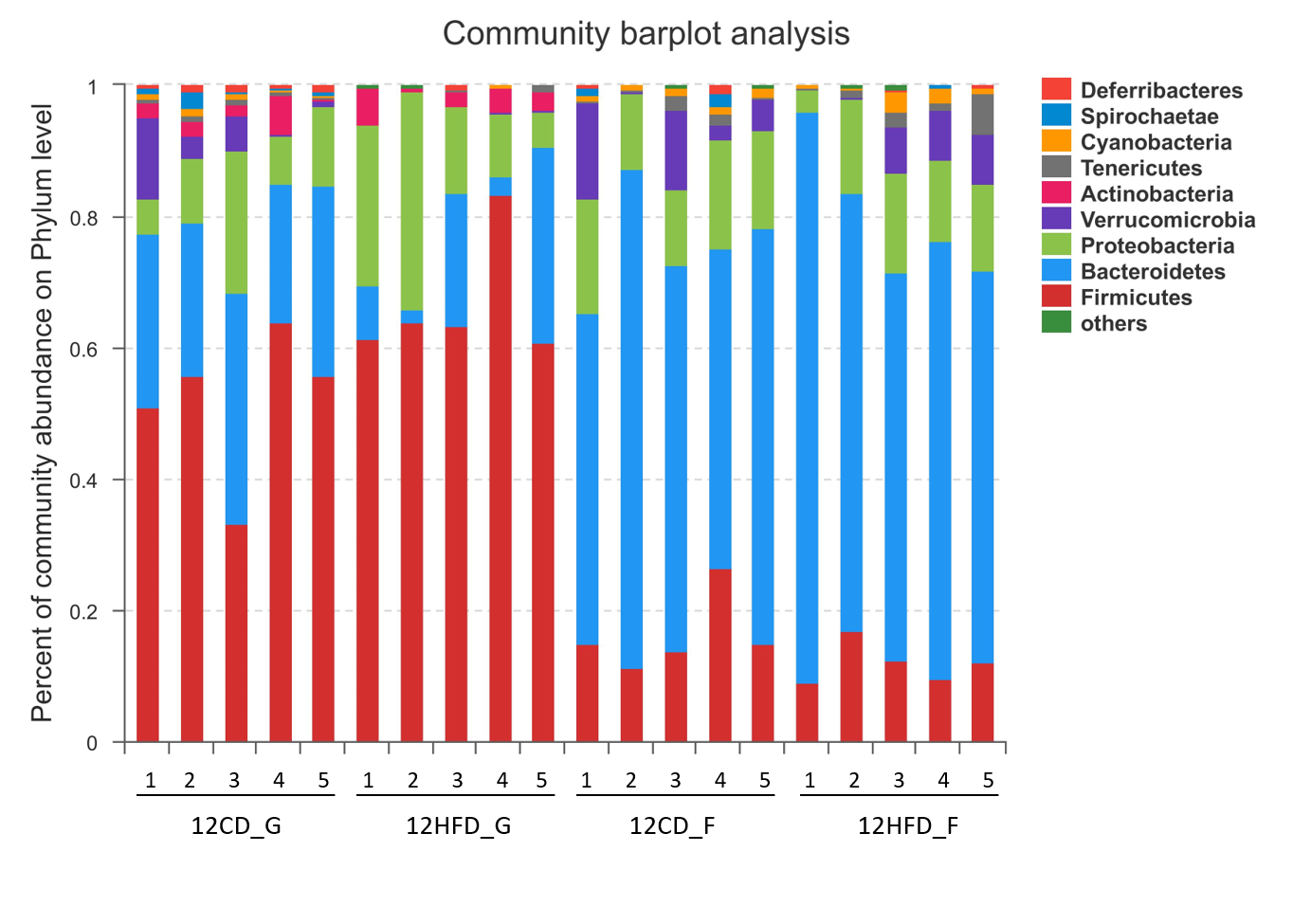

Supplement: FIGURE S2 — Bacterial taxonomic profiling at the phylum level of gastrointestinal bacteria from different groups after 12 weeks. [file Image_2.TIF]

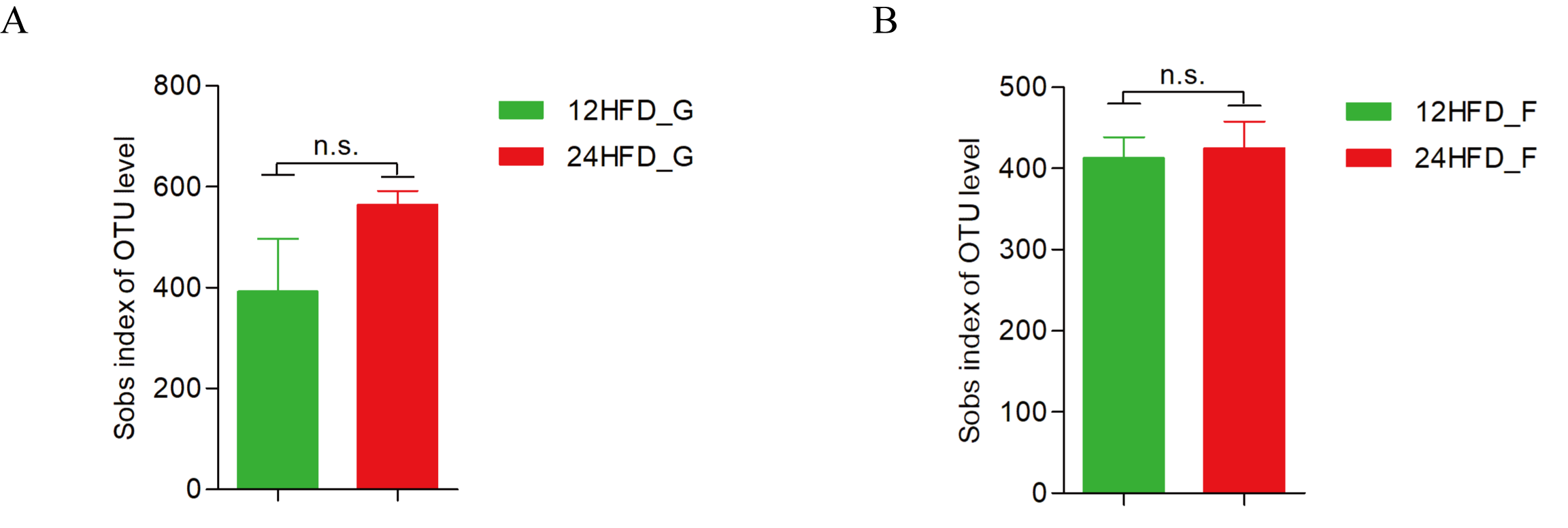

Supplement: FIGURE S3 — Alpha diversity as revealed by numbers of observed operational taxonomic units (OTUs) was not significant between 12HFD_G and 24HFD_G (A) as well as 12HFD_F and 24HFD_F (B). [file Image_3.TIF]
